# Women in ADRD: Addressing Disparities in Diagnosis and Care

**DOI:** 10.1002/alz70860_096010

**Published:** 2025-12-23

**Authors:** Tamlyn J Watermeyer, The Female Brain & Endocrine Research (FemBER) Consortium

**Affiliations:** ^1^ Edinburgh Dementia Prevention, Centre for Clinical Brain Sciences, College of Medicine and Veterinary Medicine, University of Edinburgh, Edinburgh, United Kingdom, National Institute of Health Applied Research Collaboration North East & Cumbria, Newcastle‐upon‐Tyne, England, United Kingdom, University of Northumbria, Newcastle upon tyne, England, United Kingdom

## Abstract

Women account for two‐thirds of Alzheimer's disease cases worldwide, yet remain underrepresented in many research cohorts and underserved in clinical pathways. This presentation explores the intersecting biological, sociocultural, and systemic drivers that create disparities in the diagnosis and care of women with Alzheimer's disease and related dementias (ADRD). Drawing on recent international work, I will highlight how cumulative stress, hormonal transitions, caregiver burden, and diagnostic bias compound risk and delay intervention in women—particularly among Black, Indigenous, and Global South populations. I will argue for a reorientation of dementia diagnosis and care models that are not only sex‐informed but equity‐driven. Finally, I propose a roadmap for inclusive research design, community‐based detection tools, and translational pathways that centre the biological as well as lived experiences of women across the lifespan—ensuring we diagnose earlier, care better, and prevent more equitably.

